# Nano-Biomechanical Investigation of Phosphatidylserine-Mediated Ebola Viral Attachment via Human Gas6 and Axl

**DOI:** 10.3390/v16111700

**Published:** 2024-10-30

**Authors:** Decheng Hou, Qian Mu, Weixuan Chen, Wenpeng Cao, Xiaohui Frank Zhang

**Affiliations:** 1 Department of Bioengineering, Lehigh University, Bethlehem, PA 18015, USA; kidcwp1412@sina.com; 2 Department of Biomedical Engineering, University of Massachusetts at Amherst, Amherst, MA 01003, USA; qianmu@umass.edu (Q.M.); weixuanchen@umass.edu (W.C.)

**Keywords:** Axl, Gas6, Ebola, single molecule force spectroscopy, viral entry, atomic force microscopy

## Abstract

The Ebola virus is a deadly pathogen that has been threatening public health for decades. Recent studies have revealed alternative viral invasion routes where Ebola virus approaches cells via interactions among phosphatidylserine (PS), PS binding ligands such as Gas6, and TAM family receptors such as Axl. In this study, we investigate the interactions among phosphatidylserine on the Ebola viral-like particle (VLP) membrane, human Gas6, and human Axl using atomic force microscope-based single molecule force spectroscopy to compare their binding strength and affinity from a biomechanical perspective. The impact of calcium ions on their interactions is also studied and quantified to provide more details on the calcium-dependent phosphatidylserine-Gas6 binding mechanism. Our results indicate that, in the presence of calcium ions, the binding strengths of VLP-Gas6 and VLP-Gas6-Axl increase but are still weaker than that of Gas6-Axl, and the binding affinity of VLP-Gas6 and VLP-Gas6-Axl is largely improved. The binding strength and affinity of Gas6-Axl basically remain the same, indicating no impact in the presence of calcium ions. Together, our study suggests that, under physiological conditions with calcium present, the Ebola virus can utilize its membrane phosphatidylserine to dock on cell surface via Gas6-Axl bound complex.

## 1. Introduction

The Ebola virus is a rare but deadly filovirus that was officially identified in 1976, causing Ebola virus (EBOV) disease, which, according to the World Health Organization (WHO), has a fatality rate of 25–90% [1]. EBOV outbreaks are devastating, unpredictable, and they are occurring with increasing frequency [2,3]. To date, there are six species under the genus *Orthoebolavirus* (formally *Ebolavirus*, renamed by ICTV in 2023) [4] described so far, and four of them have led to outbreaks in the past several decades. The most well-known one is Zaire ebolavirus (species *Orthoebolavirus zairense*), also referred to as the Ebola virus (EBOV), which caused the largest outbreak on record, leading to ~28,610 cases and 11,308 deaths from 2014 to 2016. It also caused the second largest outbreak during 2018–2020, causing 2287 deaths in 3470 cases [5]. Although human-infected EBOV outbreaks are regional, happening mainly in Mid and West Africa, EBOV is still an important virus that is worth studying, considering its fatality, mutability, and diverse transmission pathways. A special example is the discovery of Reston ebolavirus (species *Orthoebolavirus restonense*), which was first identified in Virginia, USA, and later found in other states in the USA and in Italy, caused by lab animals imported from Philippines [6]. No antivirals against EBOV are currently FDA-approved. A vaccine based on recombinant Vesicular Stomatitis Virus expressing EBOV glycoproteins has recently been approved by the FDA [7,8]. While the use of this vaccine is believed to help the recent outbreak, EBOV outbreaks are still poorly controlled due to civil unrest and socio-economic issues. Therefore, efforts to study EBOV are still required to inspire new therapies for future battles against the virus.

EBOV belongs to the *Filoviridae* family and is a filamentous, enveloped, non-segmented, negative-sense RNA virus [9,10]. The membrane of the Ebola virus is embedded with trimeric glycoproteins (GP) spikes. Beneath the membrane, a layer of matrix proteins (MP) that supports the membrane and secures the nucleocapsid at the center, which preserves the viral RNA together with the polymerase complex [11]. In the first step of EBOV replication, the virus attaches to the cell through interactions between cell surface receptors and the virion. This is followed by endocytosis, including micropinocytosis [12]. Subsequent trafficking of the virion to the late endosomal/lysosomal compartment results in GP proteolytic processing and GP binding to the cognate endosomal receptor NPC1. These vesicular events lead to viral-cellular membrane fusion and discharge of the viral ribonucleoprotein complex into the cytoplasm. Transcription of the negative-sense viral RNA genome by the viral polymerase complex yields mRNAs that are translated by cellular ribosomes. The buildup of viral proteins results in genome replication and the assembly of new enveloped virus particles that bud from the host cell’s surface [10,13,14], thus repeating the cycle and spreading the virus.

EBOV has an extensive tropism in the tissues they infect; macrophages and dendritic cells are considered to be their first targets. Subsequent rounds of infection follow in various cell types, including epithelial cells such as hepatocytes, stromal cells, and, to a lesser degree, endothelial cells [10,15]. Recent studies [16,17,18] indicate that a significant factor for the broad tropism of some enveloped viruses such as EBOV is the interaction between the virion membrane lipid, phosphatidylserine (PS), and cellular PS receptors in a manner that mimics apoptotic body-PS receptor interactions during the engulfment of PS-bearing dead cell debris by phagocytes. Although virus-PS receptor interactions do not serve as the only entry mechanism that enveloped viruses use, some previous findings report that this viral entry mechanism has a critical impact on viral pathogenesis [19,20]. Among all receptors transporting apoptotic cargo, TAM family receptors (Tyro3, Axl, MerTK) are potential targets for viral entry [21,22], which bind to PS after forming a complex with soluble PS-binding proteins, Gas6 or Protein S [23]. PS receptors that mediate enveloped uptake are termed phosphatidylserine-mediated virus entry enhancing receptors (PVEERs) [24]. Previous studies demonstrate that the PS-PVEER interactions are critical for EBOV pathogenesis [21,24]. Since PS-PVEER interactions are the first step in the EBOV viral entry, there is an unmet need to understand the interactions between EBOV and PS receptors biomechanically and biophysically, which could facilitate novel and rational antiviral developments.

In this study, we mainly focus on EBOV interacting with Gas6 and Axl as a potential route for viral attachment to cell surface. Gas6 is a vitamin K-dependent protein, which is initially identified in growth arrest fibroblasts. Gas6 is composed of a Gla domain, a loop region, four EGF-like repeats (EGF domain), and two laminin G-like domains (LG domain) [25,26]. The Gla domain is responsible for mediating the calcium-dependent binding to phosphatidylserine (PS), and the activation of the Gla domain requires carboxylation of its Glu residues in a vitamin K-dependent reaction, which usually undergoes in the endoplasmic reticulum during post-translational modification [25,27]. Axl is the first TAM family receptor found to interact with Gas6. It is a single-pass type 1 membrane protein, and its ectodomain consists of two fibronectin-like domains and two immunoglobulin-like domains [28]. The immunoglobulin-like domain of Axl will interact with the laminin G-like domain of Gas6 to form the Gas6–Axl bound complex [24].

Here, we will report the quantification of mechanical strengths and binding affinities among Axl, Gas6, and EBOV viral-like particles (VLP) via a custom-built atomic force microscope (AFM) based single-molecule force spectroscopy [12], which could directly measure a single bond rupture between biomolecules [29]. We also measure the binding strength between Axl and EBOV VLP in the presence of soluble Gas6 similarly by using a JPK NanoWizard 4XP (Bruker, Billerica, MA, USA) AFM to gain some knowledge that could explain the binding mechanism among PS, Gas6, and Axl by comparing all measurement results. Together, our study suggests that the Ebola virus could take advantage of the Gas6–Axl bound complex to dock on cells. The presence of calcium ions could strengthen and stabilize the virus binding to Gas6, potentially improving its infectivity and immune-escape capability.

## 2. Materials and Methods

### 2.1. Protein Constructs and Ebola Viral-Like Particles

Human Axl was purchased from Acrobiosystems (Newark, DE, USA, catalog #H5226) and was expressed from HEK293 cells, which contains AA Ala 26-Pro 449 (Accession #AAH32229). SDS-PAGE from the manufacturer determined that the purity is >95%. Human Gas6 protein was purchased from R&D systems (Minneapolis, MN, USA, catalog #885-GSB) and was expressed from mouse myeloma cell NS0, which contains AA Ala 49-Trp 678 (Accession #NP_000811). SDS-PAGE from the manufacturer determined that the purity is >90%. Several previous studies [30,31,32] successfully validated its capability in activation of Gas6-related signaling pathways, indicating that the Gla domain is biologically active. The Ebola viral-like particles (VLP) were purchased from iBT Bioservices (Rockville, MD, USA, catalog #0550-001) and were purified via dialysis to remove supplemented amino acids before the experiments. VLP’s were produced in Sf9 insect cells through infection with the recombinant baculovirus. The VLP expresses recombinant EBOV glycoprotein (GP), nucleoprotein (NP), and matrix protein (VP40), but does not contain genetic materials. An early study by Yeh et al. [33] confirmed the existence of phosphatidylserine (PS) in the Sf9 cell membrane. Therefore, PS also exists in the budded virus or viral particle envelope since the envelope is from the host cell membrane during budding. And a previous work by Ilinykh et al. also utilized this Ebola VLP to study viral trafficking to endosomes with confocal microscopy, which proves its capability in entry [34].

### 2.2. AFM Chip Functionalization, Substrate Preparation, and Biomolecule Immobilization

AFM chips (MLCT-BIO-DC, Bruker, USA) were silanized with (3-aminopropyl)-triethoxysilane (APTES). A polyethylene glycol (PEG) crosslinker, Acetal-PEG-NHS (2000 MW, Creative PEGworks, Durham, NC, USA), was connected to the APTES-coated chips and then 1 μM Gas6 was immobilized on the functionalized AFM chip according to the protocol by Dr. Hermann J. Gruber [35,36,37,38]. A previous work by Cao et al. demonstrated that the molecular weight of PEG crosslinkers has no significant impact on the measurement results [39]. The PEG crosslinker and Axl (1 μM) were linked to the amino-functionalized glass substrates (NANOCS, New York, NY, USA) using the same crosslinking approach mentioned above. The Ebola VLP was immobilized in a similar way with a 1:6 dilution from the purified stock aliquots. Loaded AFM chips and glass substrates were stored in 1× phosphate-buffered saline (PBS) at 4 °C and were consumed within 8 h to avoid degradation. Samples were prepared freshly before each experiment.

### 2.3. Single Molecule Force Spectroscopy

A single molecule force spectroscopy was conducted using a custom-built AFM and a JPK NanoWizard 4XP AFM (Bruker, Billerica, MA, USA). During the measurement, the approach speed was set to 3.76 µm/s unchanged, and the retraction speeds were set to 0.94, 1.88, 3.76, and 7.52 µm/s to achieve different unloading rates. All measurements were conducted at 25 °C in a 1× PBS or 1× PBS droplet that contains soluble non-immobilized proteins. The contact time and the indentation force between the AFM chip and the glass substrate were set to <50 ms and ~200 pN to enable the measurement of a single molecule force interaction. Minimizing the contact time is to ensure that no adhesion (rupture force) is observed between the AFM chip and substrate for the majority of contacts (67% or greater). Chesla et al. reported that assuming the binding formation obeyed Poisson statistics, a binding frequency of ~33% implies that the probabilities of forming a single, double, and triple binding bond between AFM tip and surface are 81%, 16%, and 2%, respectively, among the observed unbinding events [40]. Therefore, our experimental condition ensured that most recorded unbinding events represent the single molecule bond rupture [41].

The home-built AFM for single molecule force spectroscopy was calibrated by measuring the inverse optical lever sensitivity (InvOLS) and the spring constant of the AFM chip cantilever via thermally induced fluctuations [42]. The spring constant was calculated based on a group of force measurement curves on a non-coated glass surface. The JPK NanoWizard 4XP AFM was calibrated by following the instrument manual. The contact-based calibration was conducted to ensure the precision of force measurement. All recorded unbinding forces were corrected for the viscous drag force [43], which was obtained by multiplying the AFM chip movement velocity by the viscous drag coefficient. The viscous drag coefficient in the home-built AFM was measured by moving the AFM chip at varying velocities near the substrate and is an average of 5.05 pN⋅s/μm for the cantilever C of the AFM chip we used. The viscous drag force in the JPK NanoWizard 4XP AFM was directly measured from the data and corrected for each scanning.

### 2.4. Statistical Analysis of Single Molecule Force Measurement Results

Single molecule force measurements were conducted under different retraction speeds and at different locations. At least 100 force measurement curves were recorded at each site. The force measurement data were then analyzed by IGOR Pro or JPKSPM Data Processing software to collect unbinding forces, loading rates, and the binding frequency at each location. All unbinding forces and loading rates were grouped and analyzed by using OriginPro and were fitted using the Bell–Evans model. Data were reported as the mean and the standard deviation. Statistical analysis between groups was performed using an unpaired *t*-test by R Studio and a *p*-value less than 0.05 was considered statistically significant.

### 2.5. Microscale Thermophoresis Assay

Microscale thermophoresis (MST) measurements were performed as described [44]. Generally, Axl protein was labeled with RED-NHS (2nd Generation) dye using the Monolith Protein Labeling Kit (NanoTemper Technologies, München, Germany, catalog# MO-L011). Gas6 was 2-fold diluted in a 15-step starting from 3 μM in 1× PBS buffer supplemented with 0.01% Pluronic^®^ F-127, and was then mixed with labeled Axl (5 nM, final concentration). Mixed Axl and Gas6 samples were equally and separately loaded into 16 premium glass capillaries (NanoTemper Technologies, MO-K025). Loaded glass capillaries were placed in the reaction chamber based on the order of concentration. MST measurements were conducted using a Monolith NT.115 instrument (NanoTemper Technologies) with 100% excitation power and medium MST power at 23 °C. Seven independent replicates were analyzed to estimate the binding affinity (Kd) using the MO Affinity Analysis software (NanoTemper Technologies, version 2.3).

## 3. Results

### 3.1. Gas6 Binds to Axl with Higher Mechanical Strength and Binding Affinities Compared to PS on Ebola Viral-Like Particles

The binding strengths and affinities of Gas6–Axl and Gas6–PS on Ebola viral-like particles (VLP) are measured and evaluated via our custom-built AFM for single molecule force spectroscopy. Collected force measurement data are then analyzed to estimate the dissociation rate $k^{0}$ and reaction length γ of the interaction. $k^{0}$ describes the dissociation rate, which is used for binding affinity evaluation, and γ describes the position of the transition state, which indicates the energy barrier to form the complex. Protein molecules and viral-like particles are immobilized via an Acetal-PEG-NHS linker on APTES- or amino-functionalized surfaces. A detailed description of the methodology with schemes could be found in our previous study [45]. Briefly, measured rupture forces and loading rates are grouped and analyzed to obtain the most probable unbinding forces and the corresponding average loading rates. According to the Bell–Evans model, the relation between unbinding force and the loading rate could be described as:

(1) $$F^{*}=\frac{k_{B}T}{\gamma }ln\left(\frac{\gamma }{{k^{0}k}_{B}T}\right)+\frac{k_{B}T}{\gamma }ln(r_{f})$$

where $F^{*}$ is the unbinding force; $r_{f}$ is the loading rate; T is the temperature in Kelvin; and $k_{B}$ is the Boltzmann constant. Fitting results also provide the dissociation rate $k^{0}$ and the reaction length γ.

**Figure 1:**
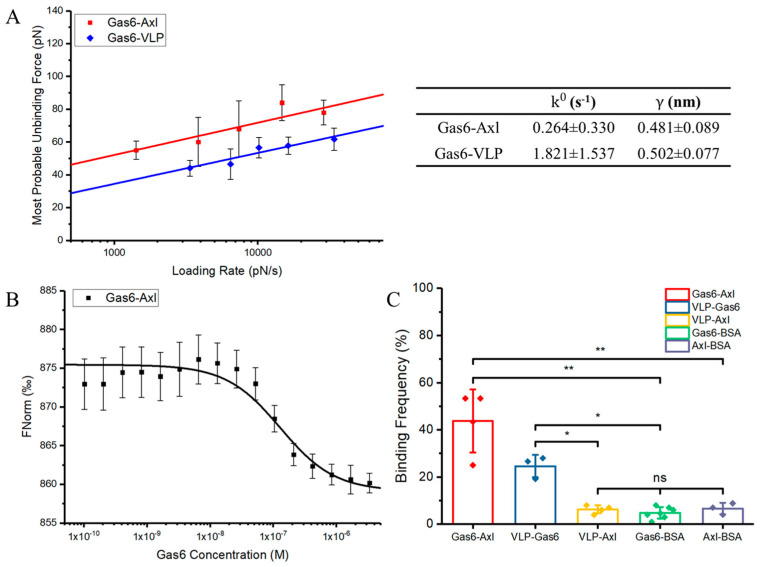
Characterization results of Gas6 interacting with Axl or PS on Ebola VLP. (**A**) Single molecule force measurement data are analyzed using the Bell–Evans model and the fitting results describe the Gas6–Axl (n = 451) and Gas6–VLP (n = 260) interactions as a relation between the unbinding force and loading rate. The fitting results also provide dissociation rates ($k^{0}$) and reaction lengths (γ) of these two interactions to evaluate their binding affinity and bound complex energy barrier, which are listed as well. (**B**) Microscale thermophoresis (MST) analysis of the interaction between Gas6 and Axl (n = 7). The fitting result is used to determine the binding affinity in the form of dissociation constant (Kd). (**C**) The binding frequency comparison is used to show the interaction specificity between experiment groups and control groups. The binding frequency measurements are all conducted under the same conditions. All given error bars show the standard deviation. Significance is determined by an unpaired *t*-test. *: <0.05; **: <0.01; ns: not significant. n is the total sample number used for analysis.

Figure 1A shows the results of our biomechanical characterization on Gas6–Axl and Gas6–VLP. Comparing these two interactions, the unbinding force between Gas6–Axl is about 20 pN stronger than the one between Gas6–VLP. Fitting results show that the dissociation rate of Gas6–Axl is 0.264 ± 0.330 s^−1^ and its reaction length is 0.481 ± 0.089 nm; the dissociation rate of Gas6–VLP is 1.821 ± 1.537 s^−1^ and its reaction length is 0.502 ± 0.077 nm. A small dissociation rate means that the bound complex has a low rate of detaching, which in turn indicates a potentially high binding affinity. Therefore, the interaction between Gas6 and Axl has a stronger binding affinity than the interaction between Gas6 and PS on Ebola viral-like particles. To ensure the interaction is specific, the binding frequency between experimental groups and control groups is measured and compared under the same conditions (Figure 1C), where the control groups are the interactions between Gas6/Axl and the bovine serum albumin (BSA) and the interaction between VLP and Axl. The results show that the binding frequency between Gas6 and Axl is about 43% on average and the binding frequency between VLP and Gas6 is about 24%. The binding frequency of all control groups is around 5%, which can be categorized as a non-specific binding. Such a difference in binding frequency indicates that most recorded interactions are specific, which are the bindings between the receptors and ligands.

We also evaluate the binding affinity of Gas6–Axl via microscale thermophoresis (MST), a traditional and reliable affinity measuring method. MST measures molecule movement in a temperature gradient to evaluate binding affinity between two molecules via fluorescence from serial diluted samples [46]. This technique is sensitive to any change in molecular properties without complicated sample preparation or immobilization, and it does not require a large quantity of samples for measurement [47]. The application of this technique in studying protein–protein interactions is widely used by many previous research [48,49,50], which makes it a well-suited alternative method to validate our findings. The response curve changing along with the Gas6 concentration is shown in Figure 1B. The MST measurement result shows that the dissociation constant (Kd) of the interaction between Gas6 and Axl is about 126 nM, which also indicates that the binding affinity between Gas6 and Axl is strong.

### 3.2. Characterization of Mechanical Strength and Binding Affinity Among Ebola VLP, Soluble Gas6, and Axl

**Figure 2:**
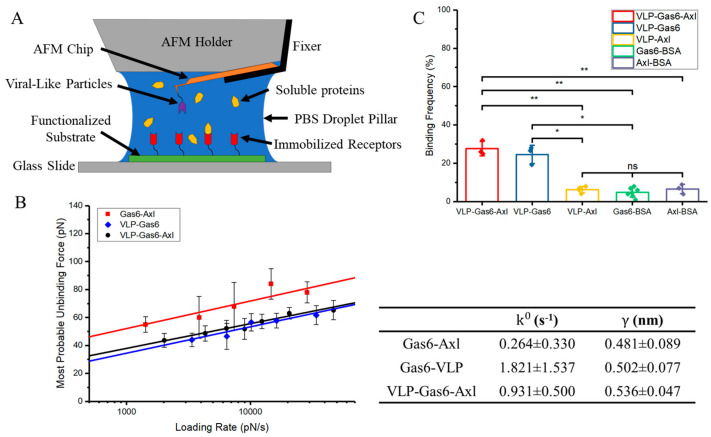
(**A**) Scheme shows the niche and the instrument settings during the measurement of VLP–Gas6–Axl (n = 427). (**B**) Single molecule force measurement result comparison between Gas6–Axl (n = 451), Gas6–VLP (n = 260), and VLP–Gas6–Axl (n = 427). Dissociation rate ($k^{0}$) and reaction length (γ) for these interactions are shown. (**C**) The binding frequency comparison is used to show the interaction specificity between experiment groups and control groups. The binding frequency measurements are all conducted under the same conditions. All given error bars show the standard deviation. Significance is determined by an unpaired *t*-test. *: <0.05; **: <0.01; ns: not significant. n is the total sample number used for analysis.

Ebola viral invasion via apoptosis mimicry is a complicated interaction that involves three types of biocomponents, which are PS on Ebola viral membrane, soluble Gas6 in microenvironment, and Axl on cell membrane surface. Our previous findings on 2-biomolecule interaction via single molecule force spectroscopy could only reveal this complicated binding procedure partially but cannot provide a more realistic binding scenario. To better understand the interaction of VLP–Gas6–Axl, we design and conduct another force spectroscopy experiment that includes all three types of biocomponents using the JPK NanoWizard 4XP AFM (Bruker, Billerica, MA, USA). Compared to the previous setup, Ebola viral-like particles (VLP) and human Axl are immobilized similarly to the functionalized AFM chip surface and the glass substrate individually. Soluble Gas6 is diluted to 1 μM in 100 μL 1× PBS, which is also used as a working droplet to generate a microenvironment for interactions among three biocomponents, shown in Figure 2A.

Measurement data are analyzed using the same method and the results are shown in Figure 2B. The fitting results show that the dissociation rate of VLP–Gas6–Axl is 0.931 ± 0.500 s^−1^ and its reaction length is 0.536 ± 0.047 nm. Similarly, the binding frequency of all experimental groups and control groups is calculated to ensure all interactions are specific. The results show that the binding frequency of VLP–Gas6–Axl and VLP–Gas6 is about 27% and 24% individually on average, while the binding frequency of all control groups is around 5% or less, which can be categorized as non-specific binding, indicating that most of the interactions detected are specific and there is no direct interaction between VLP and Axl (Figure 2C). Compared to our previous findings on Gas6–Axl and Gas6–VLP, the unbinding force of VLP–Gas6–Axl is at a similar level to that of Gas6–VLP, both are weaker than that of Gas6–Axl. Although the estimated dissociation rate of VLP–Gas6–Axl (0.931 ± 0.500 s^−1^), is smaller than that of Gas6–VLP (1.821 ± 1.537 s^−1^), the dissociation rate of Gas6–Axl (0.264 ± 0.330 s^−1^) is still the smallest. The comparison indicates that the unbinding in the VLP–Gas6–Axl system is mainly the unbinding between VLP and Gas6 under the current conditions. Therefore, in a 1× PBS environment, soluble Gas6 is preferred to bind to Axl and form a strong complex to wait for the docking of Ebola VLP since the binding affinity between Gas6 and VLP is too weak to compete against Gas6–Axl.

### 3.3. Calcium Ions Significantly Strengthen the Mechanical Strength and Binding Affinity of PS-Gas6 Bound Complex

Previous studies have shown that the calcium ions in the microenvironment also play a role in PS–Gas6–Axl bound complex [51,52]. Huang et al. studied the vitamin K-dependent proteins binding to membrane phosphatidylserine structurally with the presence of calcium ions and found that the Gla domain and calcium ions provide a unique mechanism for protein–membrane interactions [53]. Bhattacharyya et al. immobilized VSVg-pseudovirus on purified Gas6 and Protein S in a calcium-dependent manner during their study on enveloped viral entry into dendritic cells [54], which demonstrates a strong bound complex between membrane lipids and proteins. Therefore, it is necessary to study the potential impact of calcium ions on the binding affinities and strengths among Ebola VLP, Gas6, and Axl. Experiments are repeated with 1 mM calcium ions supplemented into the 1× PBS to align with the calcium concentration physiologically.

**Figure 3:**
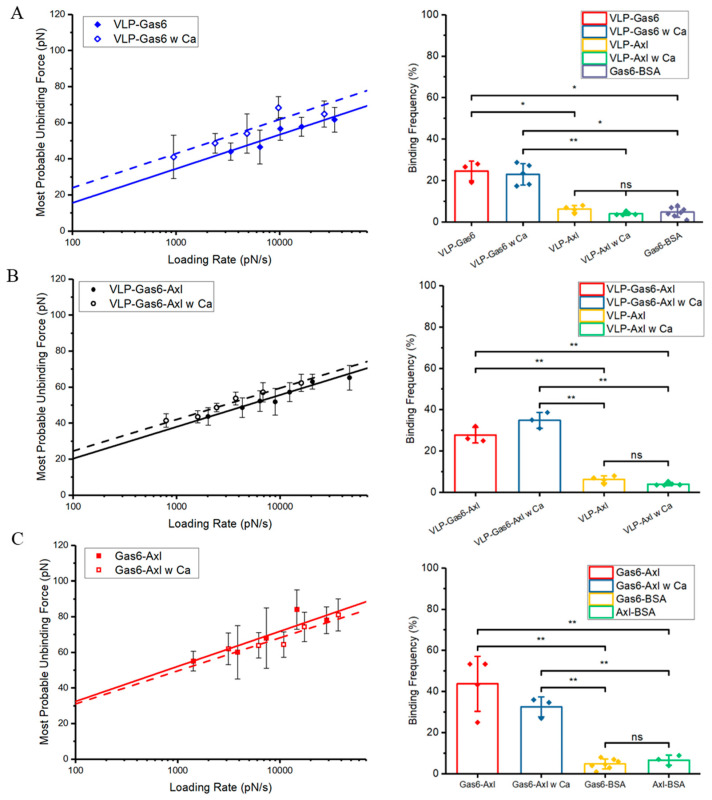
Single molecule force measurement result comparison to reveal the impact of calcium ion on interactions among VLP, Gas6, and Axl. (**A**) VLP–Gas6 interaction with (blue dashed line, n = 308) and without (blue solid line, n = 260) calcium ions present and their binding frequency comparison to examine the interaction specificity (**B**) VLP–Gas6–Axl interaction with (black dashed line, n = 397) and without (black solid line, n = 427) calcium ions present and their binding frequency comparison to examine the interaction specificity. (**C**) Gas6–Axl interaction with (red dashed line, n = 341) and without (red solid line, n = 451) calcium ions present and their binding frequency comparison to examine the interaction specificity. The binding frequency comparison is used to show the interaction specificity between experiment groups and control groups. The binding frequency measurements are all conducted under the same conditions. All given error bars show the standard deviation. Significance is determined by an unpaired *t*-test. *: <0.05; **: <0.01; ns: not significant. n is the total sample number used for analysis.

As the results show in Figure 3, in the presence of calcium ions, the unbinding forces of VLP–Gas6–Axl and VLP–Gas6 are increased compared to our previous findings, which indicates the calcium ions can improve the binding strength between PS on Ebola VLP and Gas6. This improvement does not apply to the binding strength between Gas6 and Axl. Fitting results also show that, with the calcium ion present, the dissociation rate of VLP–Gas6 is significantly improved to 0.660 ± 0.1.162 s^−1^ with a reaction length of 0.501 ± 0.146 nm, which is much smaller than the previous results, representing a strengthening on its binding affinity (Figure 3A). The binding affinity of VLP–Gas6–Axl is also enhanced to 0.523 ± 0.301 s^−1^ with a reaction length of 0.542 ± 0.056 nm, smaller than its binding affinity without the calcium ions supplemented into 1× PBS (Figure 3B). On the other hand, the impact of calcium ions on the interaction between Gas6 and Axl is very limited. Results show that the binding affinity of Gas6–Axl with supplemented calcium ions is 0.262 ± 0.515 s^−1^ with a reaction length of 0.512 ± 0.134 nm, which is comparable to its binding affinity without the calcium ion present (Figure 3C). This finding is matched to a previous study by Lew et al., who reported the binding between Gas6 and Axl is not calcium-dependent but the activation of Axl via Gas6 and PS requires the involvement of calcium ions [55]. A binding frequency measurement is conducted to ensure that recorded interactions are specific compared to the control groups.

**Figure 4:**
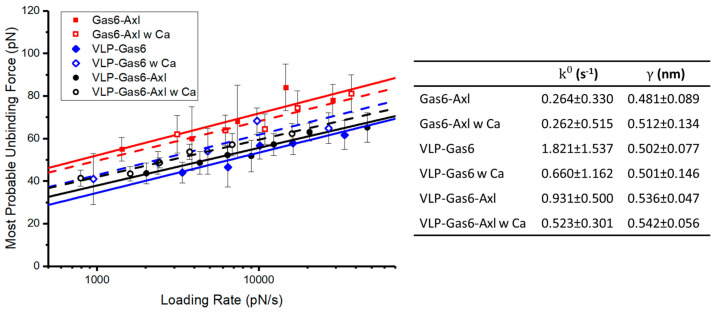
Comparison and summary of all single molecule force measurement results in this study to reveal the binding mechanism among VLP, Gas6, and Axl biomechanically and the impact of calcium ions on binding strengths and binding affinities.

With the participation of calcium ions, we compare all measurement results on these interactions to gain some new information regarding Ebola VLP binding to the Gas6–Axl complex. The comparison is shown in Figure 4. The presence of calcium ions improves the binding strength between VLP and Gas6 (5–10 pN stronger than before), but the binding strength between Gas6–Axl is still the highest and is basically not affected by the calcium ions (slightly decreased but less than 5 pN). Regarding the binding affinity comparison, without the calcium ions present, Gas6–Axl has the strongest binding affinity because its dissociation rate (k^0^) is 0.264 ± 0.330 s^−1^, much smaller than the dissociation rates of VLP–Gas6 and VLP–Gas6–Axl, which indicates a very strong binding tendency between Gas6 and Axl. In the presence of calcium ions, although Gas6–Axl still holds the strongest binding affinity and binding strength, the binding affinities of VLP–Gas6 and VLP–Gas6–Axl are enormously improved to a closed level, exhibiting a strong potential that VLP could interact with Gas6 solely, if no Axl is available in the niche. In general, the recorded unbinding activities in VLP–Gas6–Axl should majorly take place at VLP–Gas6 docking area based on our binding strength and binding affinity measurement results.

## 4. Discussion

Our study reveals that specific interactions exist between phosphatidylserine (PS) on Ebola viral-like particle (VLP) membrane and Gas6 and between Gas6 and Axl. There is no direct and specific interaction between PS on VLP and Axl. Our force spectroscopy results show that the unbinding force between Gas6–Axl is averagely 10–20 pN larger than that of VLP–Gas6 and VLP–Gas6–Axl, where the latter two are comparable to each other on the measured binding strength. The calculated dissociation rates indicate that the Gas6–Axl also has the highest binding affinity in standard 1× PBS, and Gas6–VLP has the weakest binding affinity among these groups. We then demonstrate the impact of calcium ions from the biomechanical perspective, as previous studies [51,52] revealed the unique role of calcium ions in the binding between membrane PS and vitamin K-dependent proteins, such as Gas6. In the presence of calcium ions, our findings show that the unbinding forces of VLP–Gas6 and VLP–Gas6–Axl bound complexes are increased about 5–10 pN but they are still weaker than that of Gas6–Axl. The binding affinities of VLP–Gas6 and VLP–Gas6–Axl are also significantly improved, which largely strengthens the tendency of Gas6 binding to PS on VLP but still cannot be dominant. Therefore, it is very likely that detected unbinding in VLP–Gas6–Axl mainly belongs to the VLP detaching from the Gas6–Axl bound complex.

Enveloped viral entry via PS–Gas6–Axl has been investigated for a decade. Despite the fact that many cellular level studies have revealed abundant details so far, there are still some unresolved topics that require further study. The role of Gas6–Axl as a docking site is validated by our findings and some previous research from molecular and cellular level. Morizono et al. [56] and Zhang et al. [57] reported significantly strong binding activity among Axl 293T cells, soluble Gas6, and virus/PS liposome using flow cytometry, compared to other potential PS receptors. These findings, combined with ours, indicate that the Ebola virus could attach to the cell surface utilizing Gas6–Axl complex and the involvement of Ebola glycoprotein is not necessary. Hunt et al. [58] also pointed out that the Axl could potentially enhance micropinocytosis, which could increase EBOV entry. Brindley et al. [28] later found that multiple Axl signaling pathways, such as PI3K/Akt and phospholipase C-dependent pathways, etc., could be involved to enhance Ebola viral particle uptake and the Ebola glycoprotein is not always needed for mediating viral entry. Together, the role of Gas6–Axl complex may not only work as a potential docking site for viral attachment but also play a role in enhancing viral entry.

Compared to traditional techniques for binding affinity measurements, we have discussed some features of AFM-based force spectroscopy in our previous work [45] such as establishing a 2D membrane–membrane setup to study binding affinity between biomolecules, quantifying the binding strength from mechanical and physical aspects and recording every rupture between ligands and receptors in a real-time and dynamic setting, and allowing the detection of weak interactions, etc. In this study, we have further exhibited its capability to study interactions among three biomolecules and characterize subtle differences caused by ion components in the niche, which could provide more details and new insights into interaction mechanisms.

Overall, our findings demonstrate that the Ebola virus could attach to cells via interacting with Gas6 and Axl. It is likely that soluble Gas6 will priorily dock to Axl to form the Gas6–Axl complex, which can then interact with Ebola viral particles. In the meantime, the improved binding affinity between Ebola VLP and Gas6 in the presence of calcium ions also demonstrates that Gas6 could interact with PS on viral membrane solely if lack of Axl in the niche, which provides a possibility that Gas6 could bind to PS on an Ebola viral membrane in a physiological condition. The potential of Gas6 binding to the Ebola virus could further enhance the opportunity of the virus to attach to the cell surface and could further strengthen the virus immune-escape capability with a Gas6 decoration on the virus membrane. A similar hypothesis is also proposed by Bhattacharyya et al. [54], who studied enveloped viral activation of TAM receptors using HIV-1 virions lacking any viral glycoprotein and found that these noninfectious virions could still activate TAM receptors as competent as normal pseudotype virions designed for activation purposes. In future studies, additional biomechanical investigations on interactions among other PS ligands and TAM receptors could provide more information and evidence to validate this hypothesis, offering new insights on enveloped viral entry via apoptosis mimicry and stimulating new ideas in antiviral medicine and vaccine development.

## Data Availability

Data are available upon request.
